# Supplementary material for: A platform for dissecting force sensitivity and multivalency in actin networks
Source: bioRxiv. 2023 Aug 16:2023.08.15.553463. Preprint. [Version 1] doi: 10.1101/2023.08.15.553463 (PMC10462062; doi:10.1101/2023.08.15.553463)
Supplement: Supplement 8 [file NIHPP2023.08.15.553463v1-supplement-8.pdf]

bioRxiv preprint doi: https://doi.org/10.1101/2023.08.15.553463; this version posted August 17, 2023. The copyright holder for this preprint (which was not certified by peer review) is the author/funder, who has granted bioRxiv a license to display the preprint in perpetuity. It is made available under aCC-BY 4.0 International license.

Supplemental Figures:

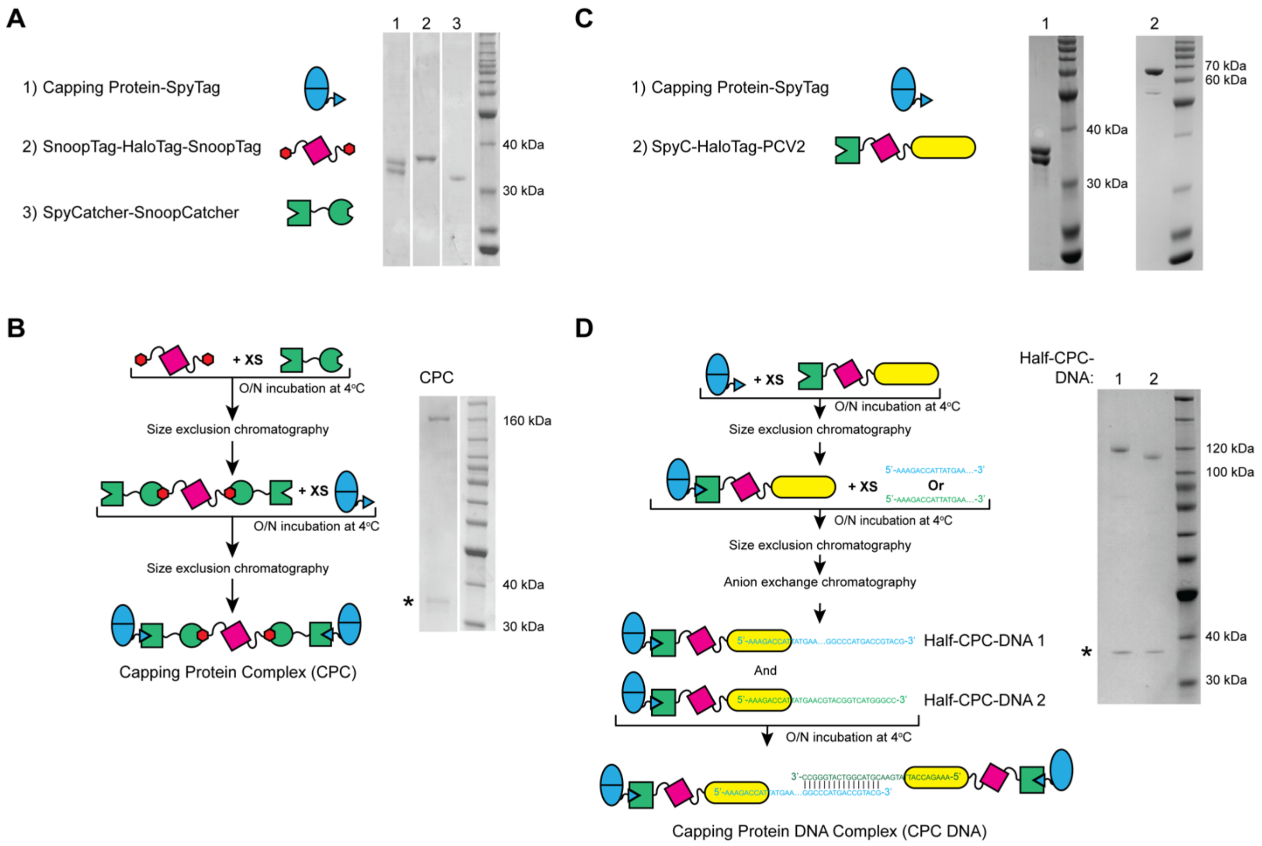

Figure S1. Assembly and purification of CPCs.
A) SDS-PAGE analysis of proteinaceous CPC components.
B) Reaction scheme and SDS-PAGE analysis of the assembled five-part CPC. Asterisk indicates CapZA, which is non-covalently associated with the CPC.
C) SDS-PAGE analysis of CPC-DNA components.
D) Reaction scheme and SDS-PAGE analysis of the two halves of the assembled CPC-DNA. Asterisk indicates CapZA.

bioRxiv preprint doi: https://doi.org/10.1101/2023.08.15.553463; this version posted August 17, 2023. The copyright holder for this preprint (which was not certified by peer review) is the author/funder, who has granted bioRxiv a license to display the preprint in perpetuity. It is made available under aCC-BY 4.0 International license.

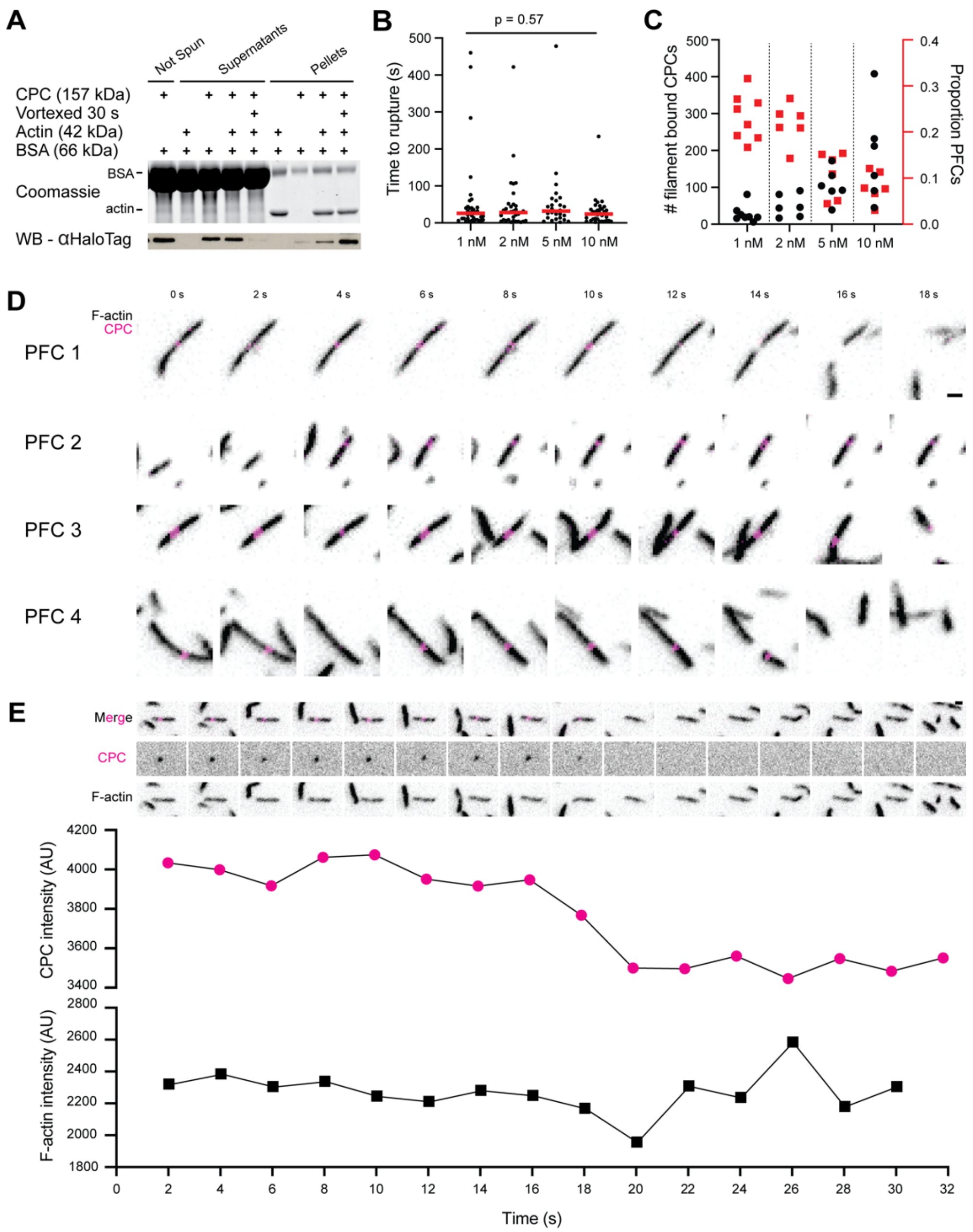

Figure S2. Characterization of PFCs assembled with CPC.

A) SDS-PAGE analysis of CPC / F-actin co-sedimentation assay. 2 mg / ml BSA was included as blocking reagent. Vortexing filaments shears them, increasing the free barbed-end concentration. Enhanced CPC co-sedimentation under this condition indicates end-dependent binding.

B) Time to rupture for PFCs from Fig. 1C, pooled by CPC concentration. Bars indicate means. n = 38 (1 nM); 33 (2 nM); 27 (5 nM); 32 (10 nM) from 26 independent trials. KW test with Dunn's correction for multiple comparisons.

C) Replotting of data from Fig. 1D, showing all data points for number of CPCs bound to filaments (black) and proportion of CPCs engaged in PFCs (red) versus CPC concentration. n = 7 (1 nM); 6 (2 nM); 6 (5 nM); 6 (10 nM) independent trials.

D) Additional montages of PFCs breaking under tension over time. Scale bar, 1 μm.

E) Top: montage where CPC bleaches prior to PFC rupture. Scale bar, 1 μm. Bottom: fluorescence intensity of F-actin and CPC in each frame. One-step bleaching of CPC indicates the presence of a single molecule.

bioRxiv preprint doi: https://doi.org/10.1101/2023.08.15.553463; this version posted August 17, 2023. The copyright holder for this preprint (which was not certified by peer review) is the author/funder, who has granted bioRxiv a license to display the preprint in perpetuity. It is made available under aCC-BY 4.0 International license.

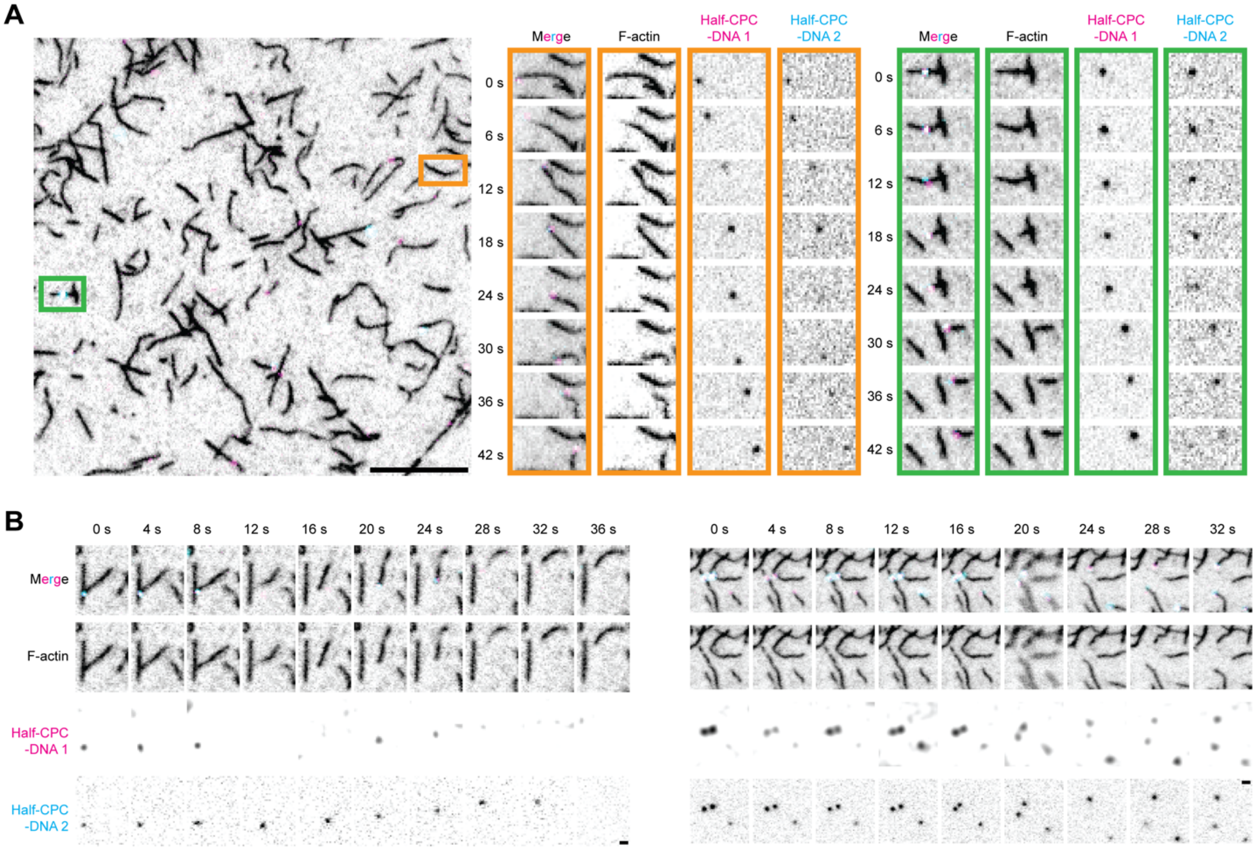

Figure S3. Characterization of dPFCs assembled with CPC-DNA.

A) Left: micrograph of a field of dPFCs prepared with CPC-DNA. Scale bar, 10 μm. Right: montage of single filament with a complete CPC-DNA attached moving over time (orange) and montage of dPFC breaking over time (green).

B) Montages of additional dPFCs breaking under tension over time. Scale bar, 1 mm.

bioRxiv preprint doi: https://doi.org/10.1101/2023.08.15.553463; this version posted August 17, 2023. The copyright holder for this preprint (which was not certified by peer review) is the author/funder, who has granted bioRxiv a license to display the preprint in perpetuity. It is made available under aCC-BY 4.0 International license.

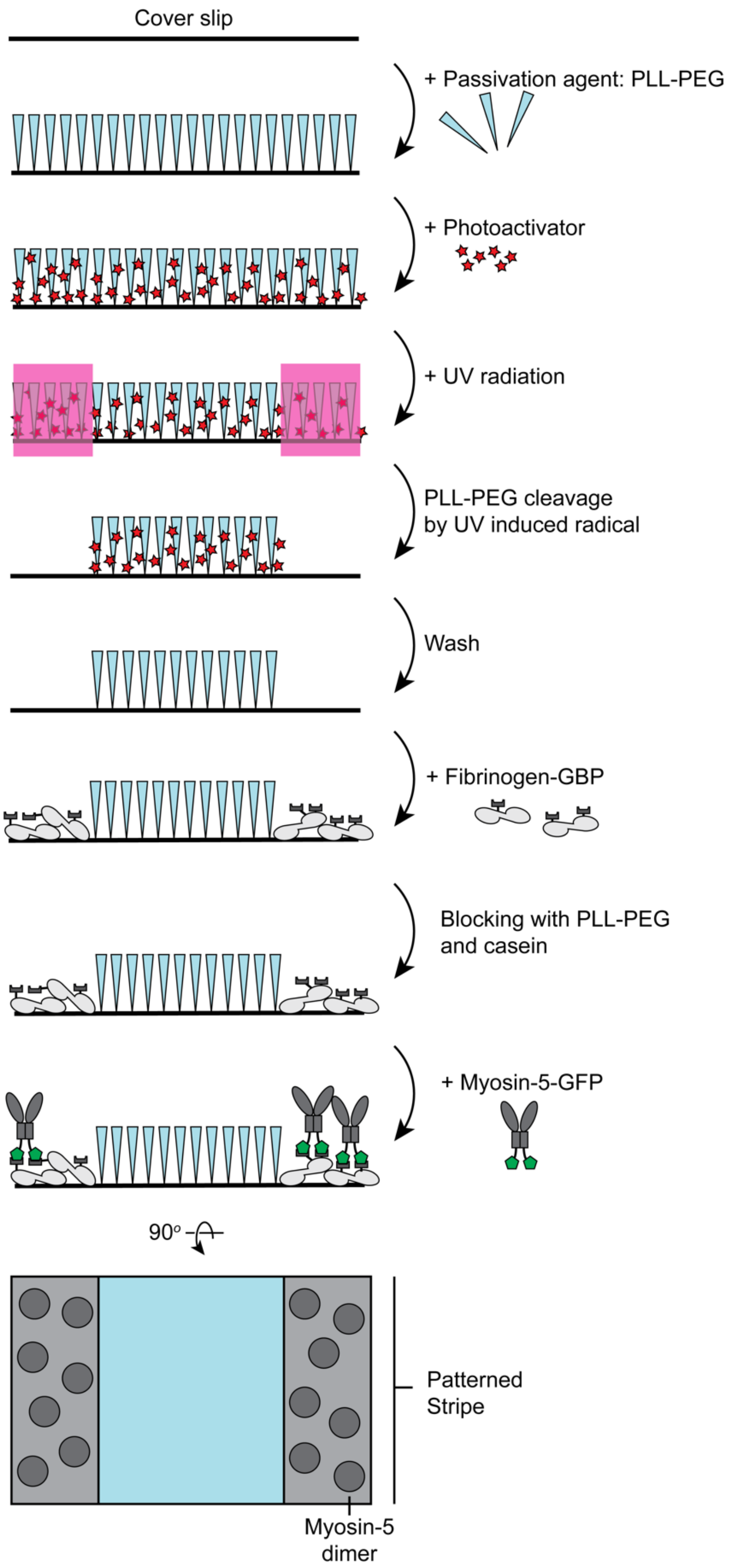

Figure S4. Protein micropatterning procedure.
Cartoon of PRIMO UV photopatterning procedure showing two adjacent stripes of myosin-5 on a coverslip (see Methods for details).

bioRxiv preprint doi: https://doi.org/10.1101/2023.08.15.553463; this version posted August 17, 2023. The copyright holder for this preprint (which was not certified by peer review) is the author/funder, who has granted bioRxiv a license to display the preprint in perpetuity. It is made available under aCC-BY 4.0 International license.

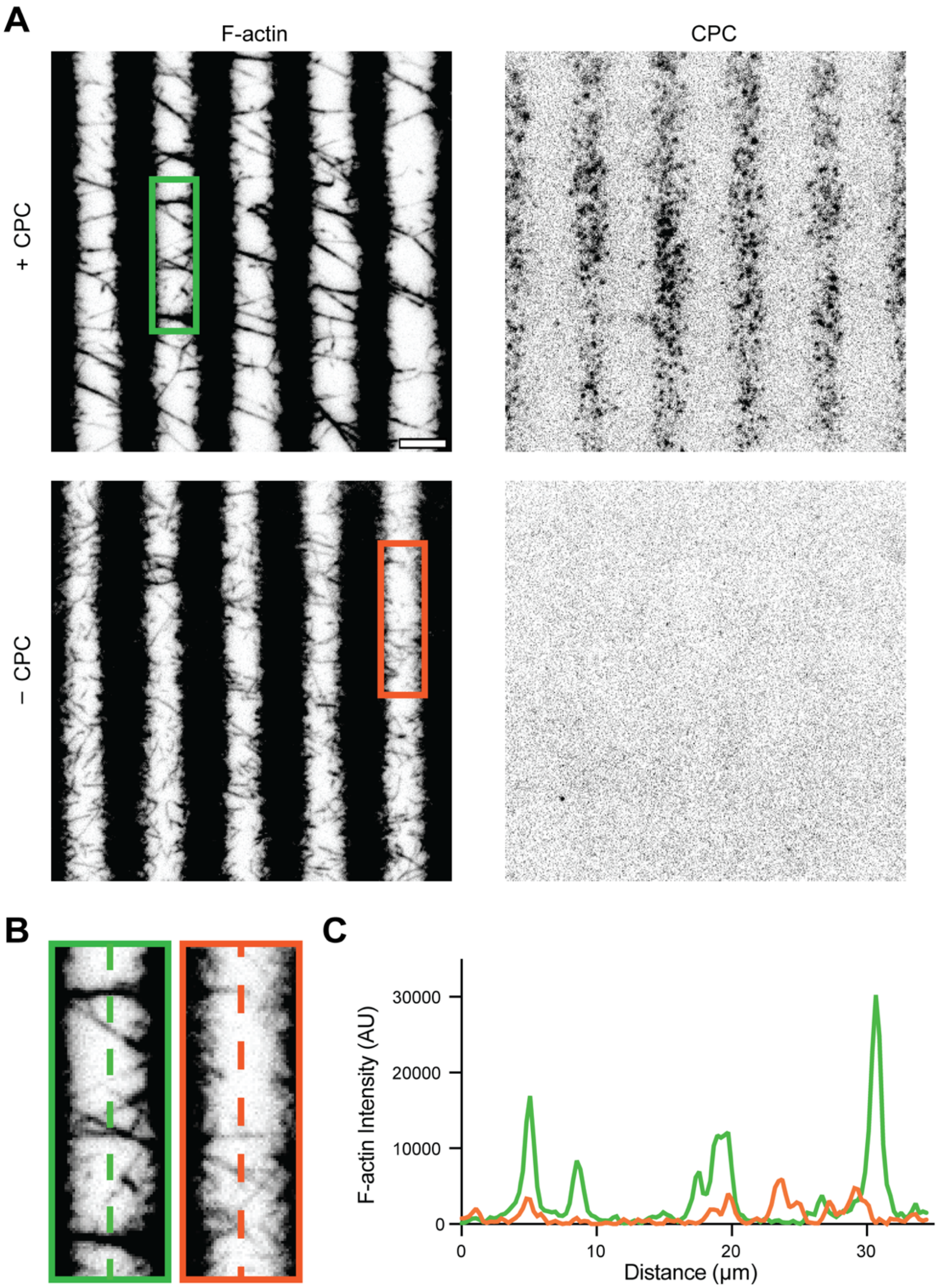

Figure S5. "Star" and "Y" configuration F-actin networks form in the presence of CPC.
A) Micrographs of F-actin on micropatterned fields of myosin-5 in the presence (top) and absence (bottom) of CPC. Scale bar, 10 μm.
B) Detail views of gaps between stripes in both conditions selected for linescan analysis (dotted lines).
C) F-actin intensity profile along linescans: + CPC condition, green; - CPC condition, orange. The lower intensity of peaks in the - CPC condition indicates reduced F-actin bundling.

bioRxiv preprint doi: https://doi.org/10.1101/2023.08.15.553463; this version posted August 17, 2023. The copyright holder for this preprint (which was not certified by peer review) is the author/funder, who has granted bioRxiv a license to display the preprint in perpetuity. It is made available under aCC-BY 4.0 International license.

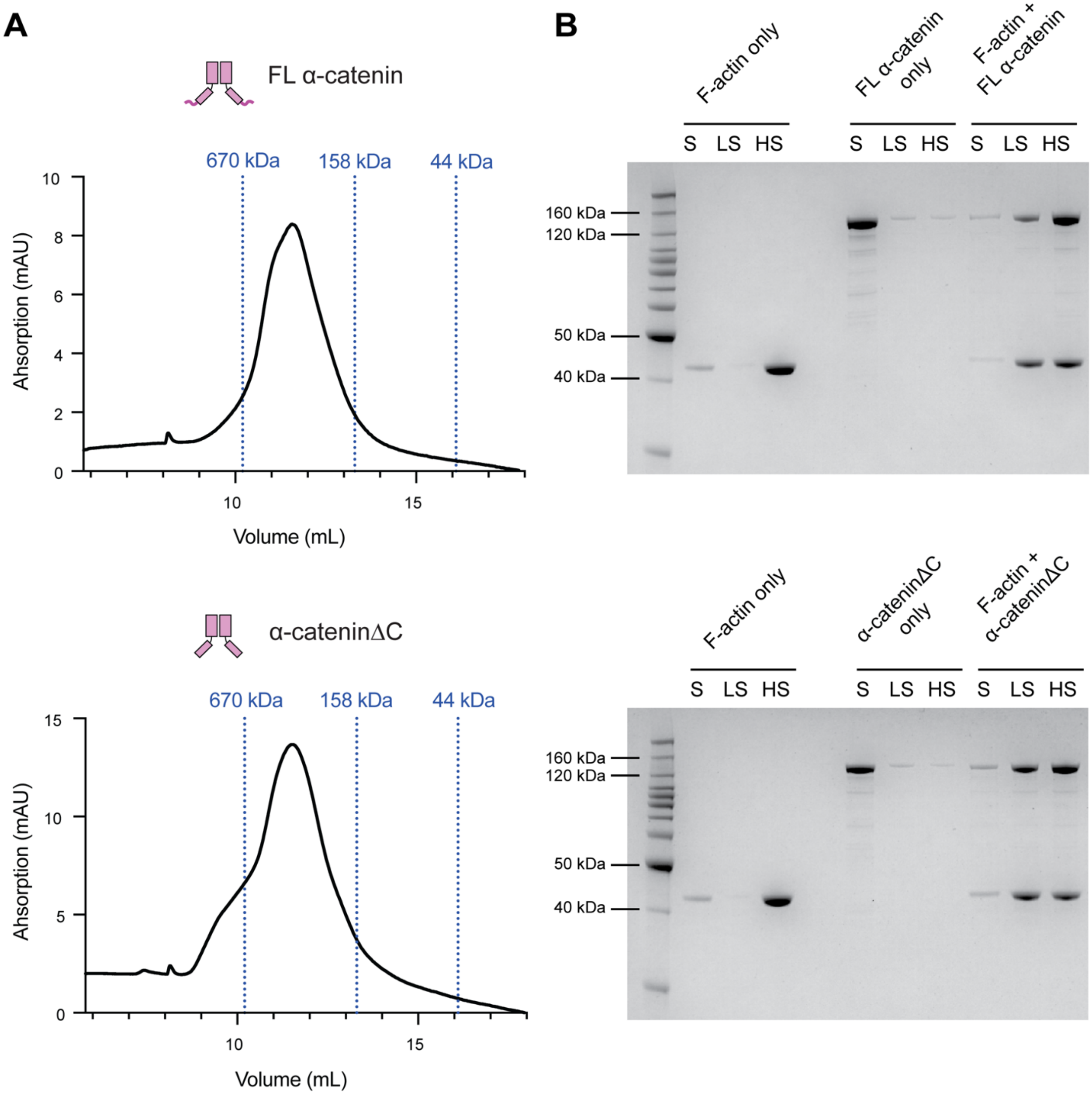

Figure S6. Purification and characterization of FL α-catenin and α-cateninΔC.
A) Size exclusion chromatography profiles of purified FL α-catenin (top) and α-cateninΔC (bottom). Molecular weights of Halo-tagged FL α-catenin and α-cateninΔC are 128 kDa and 124 kDa, respectively. Dotted lines indicate retention volumes of specified molecular weight standards.
B) SDS-PAGE analysis of co-sedimentation assays of FL αCatenin (top) and αCateninΔC (bottom). S = supernatant, LS = low speed pellet, HS = high speed pellet.

bioRxiv preprint doi: https://doi.org/10.1101/2023.08.15.553463; this version posted August 17, 2023. The copyright holder for this preprint (which was not certified by peer review) is the author/funder, who has granted bioRxiv a license to display the preprint in perpetuity. It is made available under aCC-BY 4.0 International license.

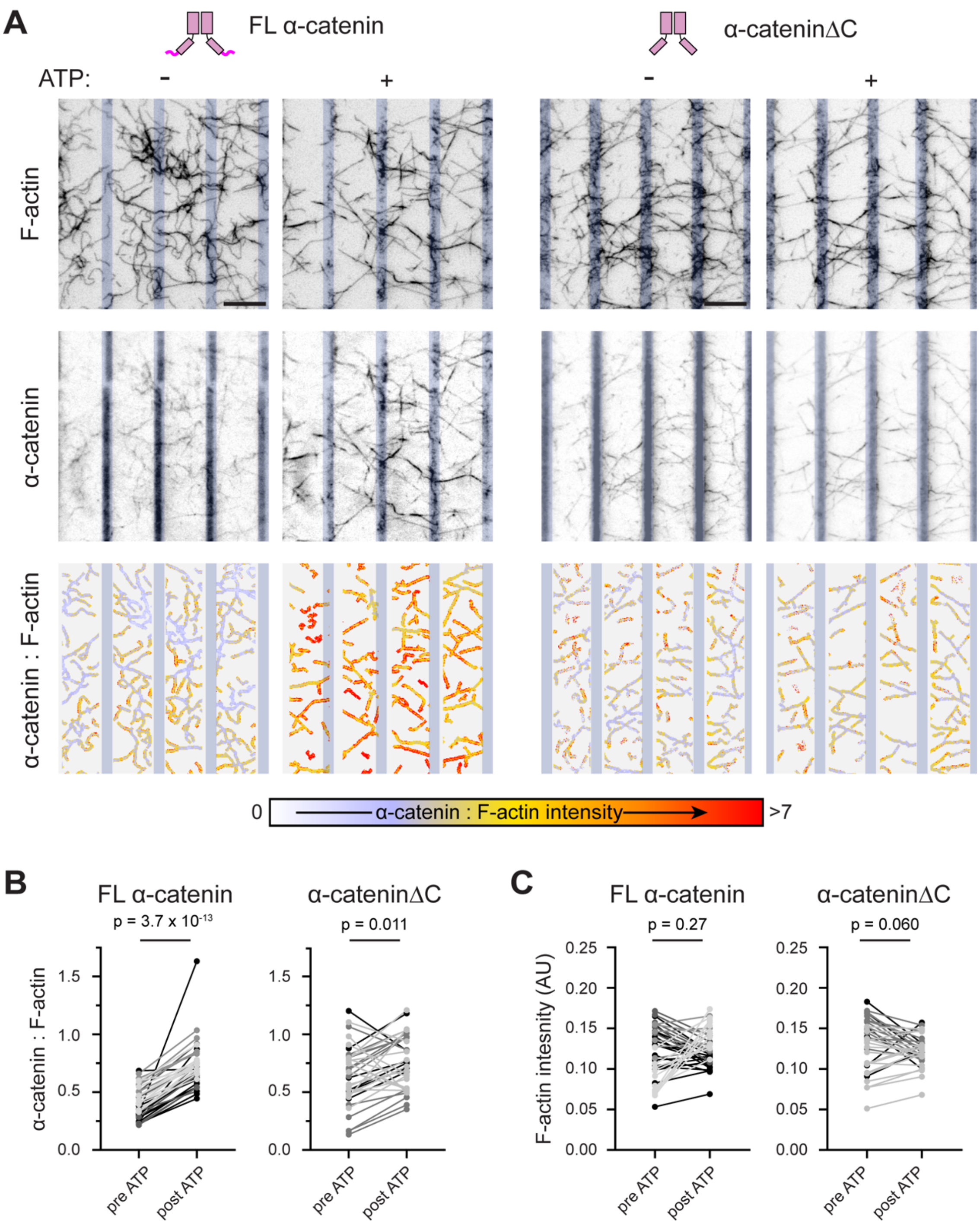

Figure S7. **Dimeric FL α-catenin features force-activated F-actin binding activity.**
A) Top: micrographs of PFC networks in the presence of FL α-catenin (left) or α-cateninΔC (right) before and after the addition of ATP. Bottom: α-catenin : F-actin fluorescence intensity ratio images of the same fields of view. Vertical bars indicate positions of myson-5 stripes. Scale bar, 10 μm.
B) Paired analysis of average α-catenin : F-actin fluorescence intensity ratio per inter-stripe gap before and after ATP addition for FL α-catenin (left) or α-cateninΔC (right). Welch's t-test: n = 40 gaps from 4 independent trials (FL α-catenin); 35 gaps from 3 independent trials (ΔC). Shades of grey indicate points from different trials.
C) Paired analysis (Welch's t-test) of actin fluorescence intensity from same data presented in **B**.

bioRxiv preprint doi: https://doi.org/10.1101/2023.08.15.553463; this version posted August 17, 2023. The copyright holder for this preprint (which was not certified by peer review) is the author/funder, who has granted bioRxiv a license to display the preprint in perpetuity. It is made available under aCC-BY 4.0 International license.

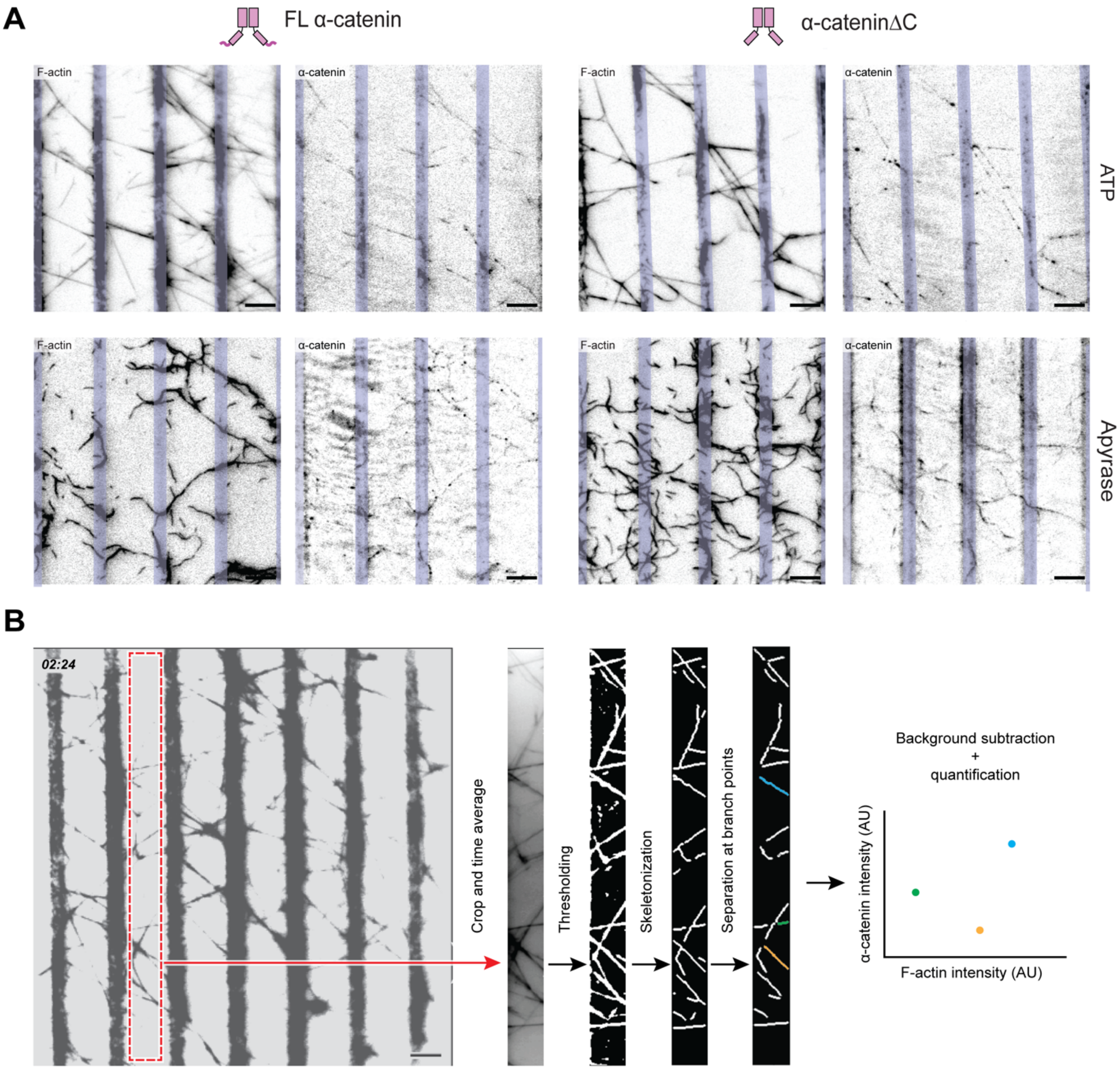

Figure S8. Image segmentation and quantification pipeline.
A) Representative micrographs of PFC networks in the presence of either FL α-catenin (left) or α-cateninΔC (right) treated with either ATP (top row) or apyrase (bottom row). Fields of view are identical to those depicted in the α-catenin : F-actin fluorescence intensity ratio images in Fig 4A. Vertical bars indicate the positions of patterned myosin-5 stripes. Scale bar, 10 μm.
B) Cartoon showing how bundle segments are identified and segmented from networks for analysis. See Methods for details. Scale bar, 10 μm.

bioRxiv preprint doi: https://doi.org/10.1101/2023.08.15.553463; this version posted August 17, 2023. The copyright holder for this preprint (which was not certified by peer review) is the author/funder, who has granted bioRxiv a license to display the preprint in perpetuity. It is made available under aCC-BY 4.0 International license.

Movie Legends:

Movie S1: PFCs prepared with CPC come under tension on a random field of myosin-5 motors. Same field of view as Fig. 1B. F-actin is black; CPC is magenta. Green arrows highlight PFCs, which turn red immediately before PFC rupture. Scale bar, 10 μm.

Movie S2: Half-CPC-DNA 1 translocates on barbed ends of actin filaments in a gliding assay. From a single field of view, left frame displays half-CPC-DNA 1 (magenta), right frame displays F-actin (black). In addition to mobile half-CPC-DNAs, some molecules adhere to the surface and do not translocate. Scale bar, 10 μm.

Movie S3: Half-CPC-DNA 2 translocates on barbed ends of actin filaments in a gliding assay. From a single field of view, left frame displays half-CPC-DNA 2 (cyan), right frame displays F-actin (black). The sample features a mixture of mobile and immobile half-CPC-DNAs. Scale bar, 10 μm.

Movie S4: dPFCs made with CPC-DNA under tension on a random field of myosin-5 motors. Same field of view as Fig. 1E. F-actin is black; half-CPC-DNA 1 is magenta; half-CPC-DNA 2 is cyan. Green arrows highlight dPFCs, which turn red immediately before dPFC rupture. Scale bar, 10 μm.

Movie S5: higher-order PFC networks under tension on micropatterned stripes of myosin-5. Same field of view as Fig. 2A. F-actin is black, CPC not shown. Regions featuring uniform high density of F-actin correspond to myosin-5 stripes. Scale bar, 10 μm.

Movie S6: FL α-catenin accumulates on PFC networks in response to tension. From a single field of view, left frame displays F-actin, right frame displays FL α-catenin. Recording was initiated after ATP addition to activate motors. Regions featuring uniform high density of F-actin and α-catenin correspond to myosin-5 stripes. Scale bar, 10 μm.

Movie S7: FL α-catenin dynamically localizes within a mechanically rearranging PFC network. From a single field of view of network analyzed in Fig. 3, left frame displays F-actin, middle frame displays FL α-catenin. Right frame displays time-averaged F-actin signal between substantial transitions assigned as states. Patterned stripes are not visible but are present to the immediate left and right of the frame. Scale bar, 2 μm.
